# The role of occlusion and micro-incontinence in the pathogenesis of penile lichen sclerosus: an observational study of pro-inflammatory cytokines’ gene expression

**DOI:** 10.1007/s11255-022-03130-7

**Published:** 2022-02-01

**Authors:** M. Czajkowski, P. Wierzbicki, A. Kotulak-Chrząszcz, K. Czajkowska, M. Bolcewicz, J. Kłącz, K. Kreft, A. Lewandowska, B. Nedoszytko, M. Sokołowska-Wojdyło, Z. Kmieć, L. Kalinowski, R. J. Nowicki, M. Matuszewski

**Affiliations:** 1 Department of Urology, Medical University of Gdańsk, Mariana Smoluchowskiego 17 street, 80-214 Gdańsk, Poland; 2 Department of Histology, Medical University of Gdańsk, Gdańsk, Poland; 3 Department of Dermatology, Venerology and Allergology, Medical University of Gdańsk, Gdańsk, Poland; 4 Division of Medical Laboratory Diagnostics, Medical University of Gdańsk, Gdańsk, Poland; 5 Greater Poland Cancer Center, Poznan University of Medical Sciences, Poznań, Poland; 6 Molecular Laboratory, Invicta Fertility and Reproductive Center, Sopot, Poland

**Keywords:** Penile lichen sclerosus, Micro-incontinence, IL-1, IL-6, IFN-γ, TGF-β1

## Abstract

**Purpose:**

To assess the expression of selected cytokines in penile lichen sclerosus (PLS) and associate them with the occurrence of micro-incontinence (MI) in different stages of PLS.

**Methods:**

The skin biopsies from 49 PLS affected, and 13 from nonlesional foreskins (healthy control adult males undergoing circumcision due to phimosis caused by short frenulum) were obtained. All specimens were used for RNA extraction and RT-qPCR. Quantitative assessment of the gene expression of *interleukin 1-A (IL-1A*), *interleukin 1-B (IL-1B)*, *interleukin 1 receptor antagonist (IL-1RN), interleukin 6 (IL-6), transforming growth factor β1 (TGF-β1), and interferon-gamma (INF-γ)* was performed. To determinate the presence of MI, the patients were asked about voiding patterns, especially leaking tiny drops of urine from the urethral meatus after urination.

**Results:**

IL-1A, IL-6, and INF-γ mRNA levels were approximately 150, 16, and 59 times higher in PLS than in control samples, respectively. The highest IL-1A mRNA levels were observed in early PLS (*n* = 13), INF-γ in moderate PLS (*n* = 32), while IL-6 in severe PLS (*n* = 4). MI was noted in 45 PLS patients vs. 0 in control (*p* < 0.0001). IL-1A and IL-6 vs control ratios were concentration (ca.) 400 and 30 times higher, respectively, in MI PLS samples than in PLS without MI.

**Conclusion:**

Occlusion and irritating urine effect are associated with the clinical progression of penile LS with increased mRNA expression of IL-1A, INF-γ, and IL-6 pro-inflammatory cytokines in the foreskin.

## Introduction

Penile lichen sclerosus (PLS) is a kind of chronic and fibrotic dermatosis. The typical clinical manifestations are white plaques and induration which can appear at every part of penile skin or mucosa. Less common symptoms are telangiectasias, purpura, and nonspecific hypopigmented or erythematous macules. The most often localization is prepuce, frenulum, penile glans, and urethral meatus. Sclerosis seems to be the main factor that provides to PLS complications such as phimosis, paraphimosis, painful erections, dyspareunia, and urethral strictures [1]. The most frequently postulated risk factors associated with PLS development are: lack of circumcision, all kinds of genital skin injury (genital piercings, friction during sexual intercourse, and surgery), elevated mean body mass index (BMI), diabetes mellitus, and post-micturition micro-incontinence (MI) [1, 2].

The true prevalence of PLS is unknown mainly due to the lack of histopathological examination performed in every case of suspected PLS or asymptomatic course of the disease. There are some studies of the prevalence of LS in foreskin specimens obtained during circumcision, and there is a large discrepancy in data collected in this research, ranging from 1 to 67.4% [3, 4].

The etiology and pathogenesis of PLS are obscure. The most frequent postulated hypotheses are infectious etiology, autoimmune disease, and chronic irritation [5].

However, the literature provides insufficient information about the influence of the irritating urine effect in occlusion conditions. Moreover, there is a lack of data concerning the pathomechanism involved in PLS development.

Our study aimed to assess the mRNA expression of cytokines such as interleukin 1-A (encoded by *IL1A* gene, alias *IL-1A*), interleukin 1-B (*IL1B, IL-1B*), interleukin 1 Receptor Antagonist (*IL1RN, IL-1RN*) interleukin 6 (*IL6, IL-6*), transforming growth factor β1 (*TGFB1, TGFβ-1*), and Interferon-gamma (*INFG, INF-γ*) in PLS. In addition, we tried to find out if occlusion and MI could be considered as triggering factors for the production of pro-inflammatory cytokines in PLS. Moreover, samples of healthy preputial skin were included in the study for comparison.

## Materials and methods

### Patients and skin biopsies

Skin biopsies from foreskin were obtained from 62 (49 PLS, and 13 healthy control) adult males undergoing circumcision at the tertiary referral Department of Urology, between January 2017 and December 2019. Control patients were a group of healthy adults, who underwent surgery due to a short frenulum that provides phimosis. Histological examination of whole specimens from this group confirmed unchanged skin, while biopsies from other groups confirmed PLS. In addition, the patients suffering from PLS were divided into three groups: early, moderate and severe PLS. The allocation to individual PLS staging groups depended on the histopathological classification described in the part *Histological pattern of LS.* To determinate the presence of micro-incontinence, the patients were asked about voiding patterns before phimosis formation, especially leaking tiny drops of urine from the urethral meatus after urination. Moreover, to exclude a systemic inflammatory response in all patients, C-reactive protein (CRP) levels were measured before surgery. An independent Bioethics Committee has approved the present study (decision No. NKBBN/369/2017), and all patients had signed written informed consent before surgery.

### Skin biopsies’ acquisition

All skin biopsies were obtained during complete circumcisions that were operated by the same urologist using the sleeve circumcision technique. The indication for surgery was phimosis (*n* = 62; 100%). Patients had not received topical treatment for 6 months before circumcision. After circumcision, tissue fragments (foreskin) were cut into two similar fragments; one was immediately placed in 5 volumes of RNA-Later (Ambion Inc., a brand of Thermo Fisher Scientific, Inc.), and stored in a fridge for 6 to 24 h, followed by storage in − 80 °C until further processing (RNA extraction). The remaining tissue fragment underwent fixation in ~ 10 volumes of buffered 4% formaldehyde (pH = 7.4, POCH, Poland), and stored at 4 °C. Formalin-stored tissues were further processed for histopathological assessment.

### Assessment of the mRNA expression of IL-1A, IL-1B, IL-1RN, IL-6, TGF-β1 and IFN-γ genes

RNA isolation was optimized by adapting a modified method of Chomczynski and Sacchi [6] using a Total RNA Mini protocol isolation kit (A&A Biotechnology, Poland). Briefly, RNA-Later samples were defrosted, drained of liquid with a sterile paper towel; 3 × 3 × 3 mm tissue fragment was cut out for RNA extraction. The remaining tissue sample of similar size was placed in a sterile vial and immediately placed in liquid nitrogen, and then was stored at − 80 °C. The processed biopsy tissue was cut with sterile scissors to as small as possible fragments and placed in a 1.5 ml Eppendorf tube with 800 µl Fenozol. The tube was incubated in TS-100C (BioSan, Latvia) thermoblock at 50 °C for 45 min. After adding 200 µl chloroform (POCH), samples were gently mixed, and incubated at room temperature (RT) for 5 min, followed by centrifugation at 12,000 rpm for 15 min at 4 °C. The next steps of RNA extraction were carried out by the manufacturer’s protocol with the final elution volume of 100 µl RNAse-free water. After RNA quantity and purity assessment (Epoch 800 plate reader), RNA was stored at − 80 °C for further analyses. cDNA synthesis was performed as previously described [7]. Total RNA samples (2 µg) were reverse transcribed with RevertAid Reverse Transcriptase (Fermentas; Thermo Fischer Scientific, Inc.). Details concerning the qPCR methodology are provided in Table 1. 1 µl of four times diluted cDNA was used in 10 µl total volume of qPCR reaction. All reactions were run in duplicate; the measurement of glucuronidase beta (GUSB) gene expression was used for the normalization of qPCR results with Livak and Schmittgen’s 2ΔΔCq method [8, 9].

**Table 1:** Details of qPCR assays

Gene name	GeneBank transcript Acc. Number	Primers’ sequences	qPCR reaction conditions	qPCR reaction content
*IL-1α*	NM_000575.4	5’-TAGGTCAGCACCTTTTAGCTTC 5’-GTATCTCAGGCATCTCCTTCAG	95 °C, 3 min; 45x (95 °C, 5 s; 59 °C, 10 s; 72 °C, 10 s; 75 °C, 10 s—sample reading) Melting curve: 95 °C, 15 s; 60 °C, 1 min; 60 °C → 95 °C reading every 0.3 °C	5 µl AmplifyMe NoRox SybrGreen (with SybrGreen fluorophore) (Blirt, Poland), 200 nM each primer, Σ 10 µl
*IL-1β*	NM_000576.2	5’-CCTTAGGGTAGTGCTAAGAGGA 5’-TACAGACACTGCTACTTCTTGC		
*IL-1RN*	NM_173841.2	5’-GGCACTTGGAGACTTGTATGAA 5’-GAGCTGAAGTCACAGGAAGTAG		
IL-6	NM_000600.4	5’-CACTCACCTCTTCAGAACGAAT 5’-AGGCAAGTCTCCTCATTGAATC		
*INF- γ*	NM_000619.2	5’-TGGAAAGAGGAGAGTGACAGAA 5’-TATTGCTTTGCGTTGGACATTC		
*TGF-β1*	NM_000660.6	5’-GAGCTGTACCAGAAATACAGCA 5’-AACTCCGGTGACATCAAAAGAT		
*GUSB*	NM_000181.4	5’-ATGCAGGTGATGGAAGAAGTGGTG 5’-AGAGTTGCTCACAAAGGTCACAGG		

### Statistical analysis

Statistical analysis was performed using GraphPad Prism ver. 6.07 (GraphPad Software) software. The following statistical tests were used: 2 × 2 Fisher’s exact, Shapiro–Wilk normality; parametric Student *t*- and non-parametric Mann–Whitney *U*, Wilcoxon signed-rank, Spearman’s correlation tests. A two-sided *p* < 0.05 was considered to indicate a statistically significant difference, with a 95% confidence interval in all analyses.

## Results

### Patient characteristics

Based on the histopathological examination, the studied patients are divided on four groups: control skin (*n* = 13; 21%), early PLS (*n* = 13; 21%), moderate PLS (*n* = 32; 52%), and severe PLS (*n* = 4; 6%).

The 43 PLS patients (69%) reported post-micturition MI (early LS *n* = 9; moderate LS *n* = 32; severe LS *n* = 2), while 4 PLS (6%), and all control patients did not report any problems with micturition. Moreover, two patients with severe PLS suffered from urinary incontinence after radical prostatectomy.

Demographic, clinical, and histopathological data of the patients established by uropathologist were recorded in the database. There were no differences in body mass index (BMI) or C-reactive protein (CRP) concentrations between the studied patients’ groups (Table 2).

**Table 2:** Demographic characteristic and histopathological diagnosis of patients

Group	N	Age [y]: mean ± SD; median (range)	P* (LS grades)	*P* (all groups)	BMI: mean ± SD	P* (LS grades)	P* (all groups)	CRP [mg/dL]: mean ± SD	P* (LS grades)	P* (all groups)
Control	13	31.38 ± 14.80; 24.0 (21–65)		0.0011	28.21 ± 6.96		Ns (0.86)	1.76 ± 2.85		Ns (0.11)
LS	49	53.20 ± 17.61; 50.0 (23–86)			27.90 ± 4.93			4.62 ± 9.88		
LS early	13	54.92 ± 18.82; 55.0 (23–86)	Ns (0.14)		27.58 ± 7.11	Ns (0.93)		5.27 ± 9.82	Ns (0.76)	
LS moderate	32	50.63 ± 16.65; 46.0 (23–83)			27.58 ± 7.11			4.60 ± 10.59		
LS severe	4	65.25 ± 17.21; 71.0 (45–86)			27.58 ± 7.11			2.70 ± 3.14		

*Student’s *t*-test used if Shapiro–Wilk normality test was passed, if not—Mann–Whitney *U* test was applied

During the follow-up period (min–max: 18–60 months), there was no recurrence of penile LS.

### Expression of the cytokines’ genes in PLS versus control group

**Fig. 1:**
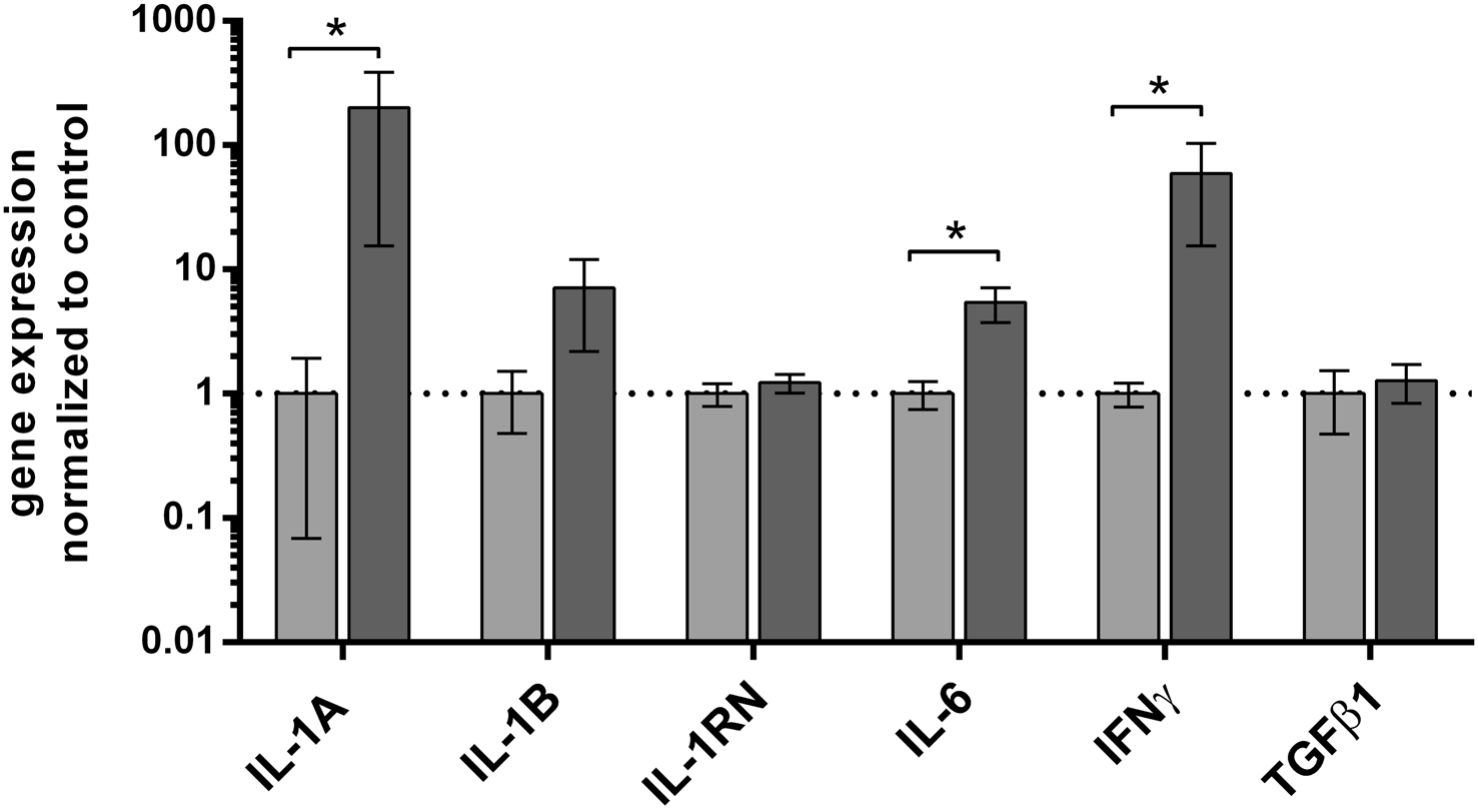
Cytokines’ gene expression at the mRNA level in foreskin samples of penile lichen sclerosus and control patients. Gene expression was assessed as described in methods. The ordinate axis is shown on a logarithmic scale. Bars and whiskers represent the mean ± standard deviation of the mean (SEM) of genes’ expression levels in PLS (dark grey bars) normalized to control foreskin (light grey bars) samples (presented as 1; dotted horizontal line at 1). *P < 0.05 (Mann–Whitney *U* test between each group, solid line). *PLS* penile lichen sclerosus

First, we checked the expression pattern of interesting genes in PLS without dividing them into groups by severity in comparison to control foreskin samples. The results are shown in Fig. 1. We found the highest level of *IL-1A* gene expression in PLS, with concentration (ca.) 150 times higher than in control foreskin samples (*p* < 0.05). Furthermore, we observed that levels of *IL-6* and *IFN-γ* mRNA were seven and 59 times up-regulated in PLS in comparison to control samples (*p* < 0.05). There was no difference in expression of other analyzed cytokines’ genes between PLS and control samples, as presented in Fig. 1.

### The expression of cytokines’ genes in lichen sclerosus penile tissues divided by stages

**Fig. 2:**
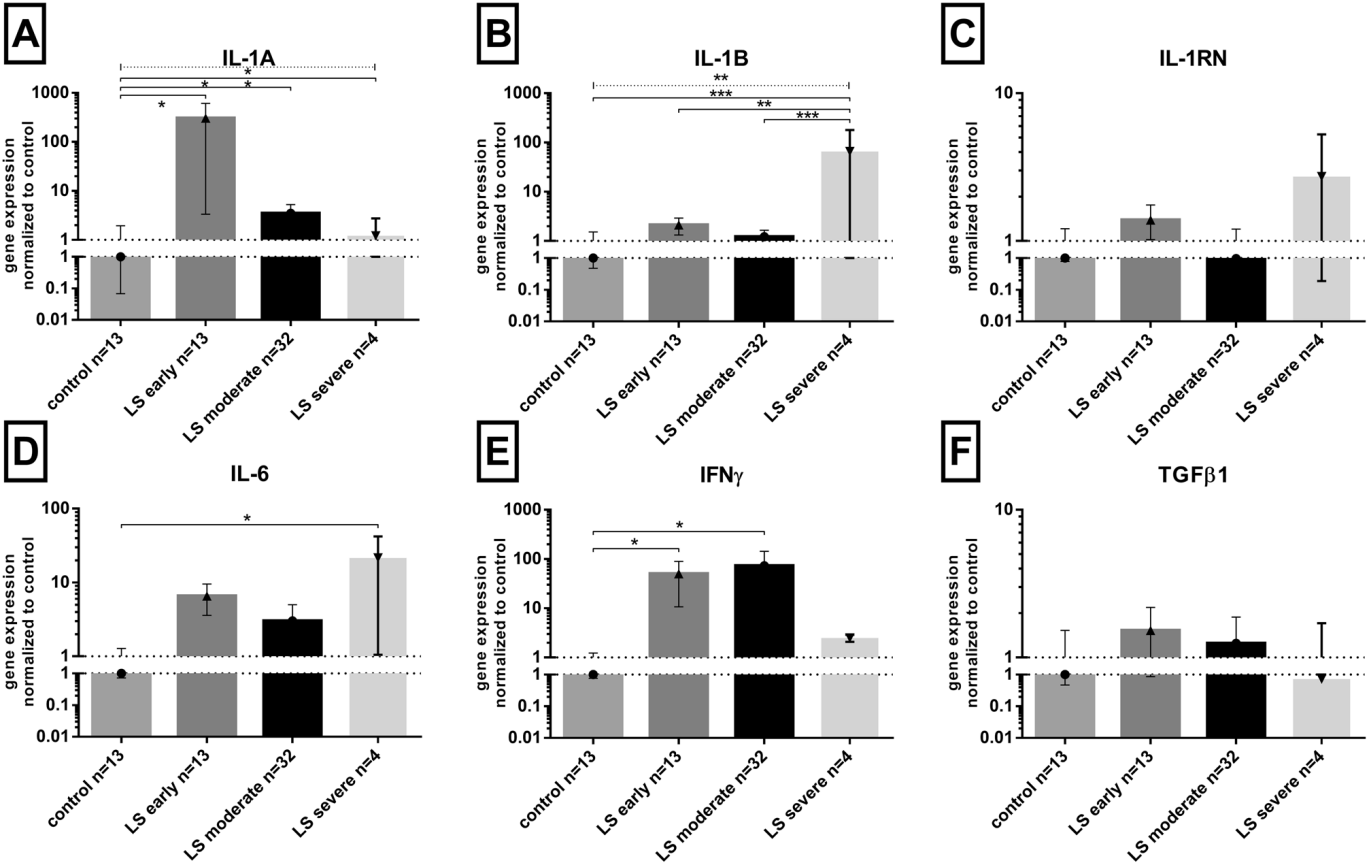
Cytokines’ mRNA levels in penile lichen sclerosus stages in relation to control samples. Gene expression was assessed as described in “Materials and methods”. The ordinate axis is shown on a logarithmic scale. Bars and whiskers represent the mean ± SEM normalized to control foreskin samples (presented as 1). *P < 0.05, **P < 0.01, ***P < 0.001 (Mann–Whitney *U* test between each group, solid lines above bars; Kruskal–Wallis ANOVA test between all groups, dotted line above bars). *LS* lichen sclerosus

The expression of the cytokine’s genes in PLS patients according to the clinical stage of the disease is shown in Fig. 2. The highest expression of *IL-1A* gene was noted in early PLS (306 times higher than in controls, whereas in four patients with moderate and severe PLS, the increase was only approximately 4- and 1.5-fold, respectively (Fig. 2A). Similarly, the highest expression of *IFN-γ* gene (Fig. 2E) was found in early and mild PLS cases (ca. 50 and 73 times higher than in controls). On the contrary, the highest levels of *IL-1B* (Fig. 2B), and *IL-6* (Fig. 2D) mRNAs were found in severe PLS (ca. 65 and 22 times higher than in control, respectively). There were no differences in the expression of *IL-1RN* (Fig. 2C) and *TGFβ1* (Fig. 2F) genes between PLS and control patients.

The statistical analysis revealed a weak (R2 = 0.31, *P* = 0.028) positive correlation between IL-6 mRNA levels in PLS patients inflamed tissues, and the patient’s age (Spearman’s non-parametric test). The IL-6 mRNA levels shared a very weak positive correlation with disease progress (R2 = 0.14, *P* = 0.043, plot not shown). We also observed a weak negative correlation between IL-1B mRNA levels in PLS samples, and BMI ratio (R2 = − 0.29, *P* = 0.04). Plots were not shown for the correlation analyses.

There was no correspondence between cytokines’ mRNA levels, and C-reactive protein (CRP) serum concentration (plot not shown).

### Histological pattern of LS

**Fig. 3:**
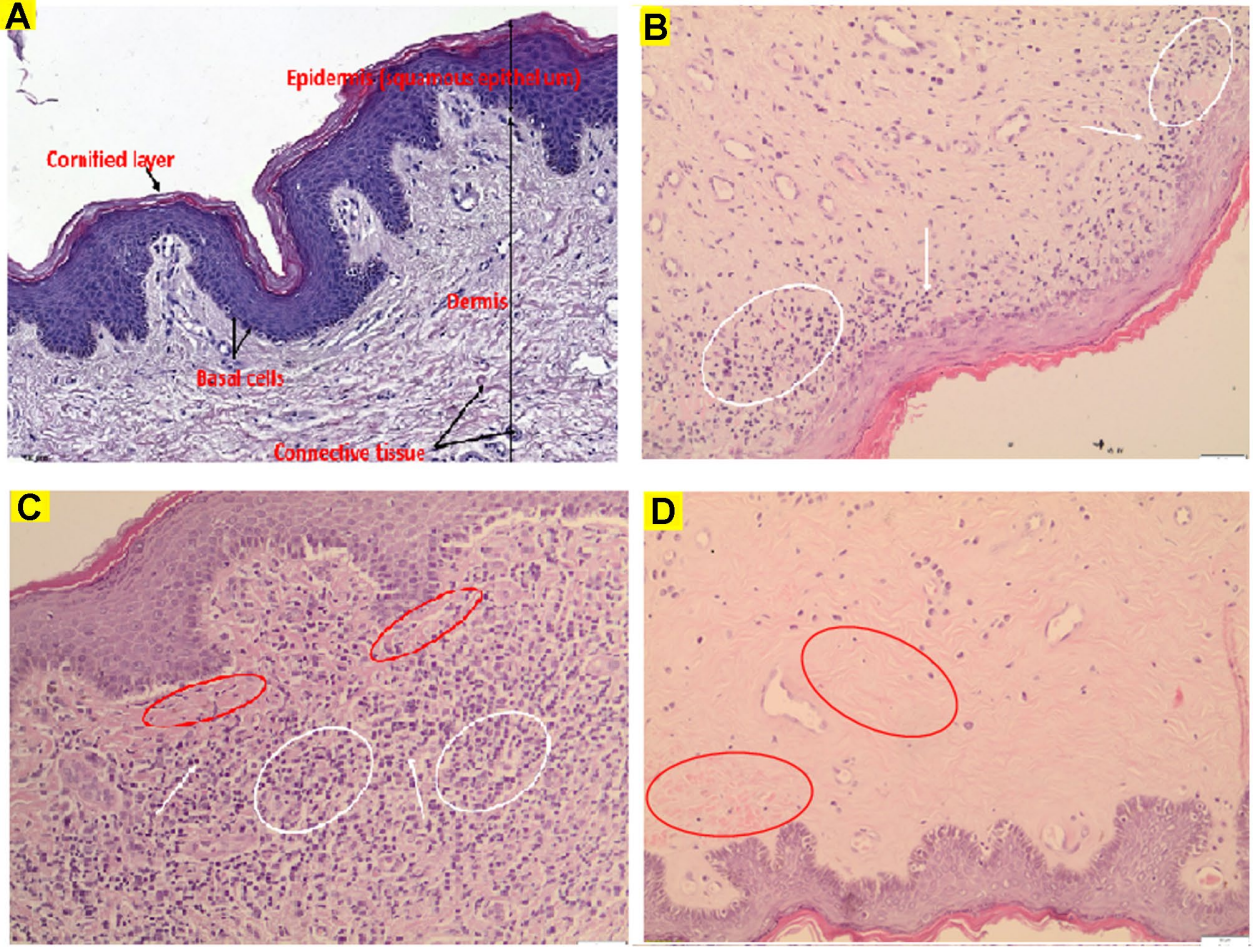
Representative microphotographs of normal foreskin and LS histological sections. **A** Normal control foreskin. **B**–**D** Early, moderate and severe LS stages, respectively. Symbols for **B**–**C**: white arrows—lymphocytes, white ellipses—dense inflammatory infiltrate, red ellipses—dermal hyalinization. Hematoxylin and eosin staining, scale bars represent 100 μm for (**A**) and 50 μm for (**B**–**E**)

Representative microphotographs of PLS biopsies as compared to normal foreskin samples are shown in Fig. 3. Almost any mono- and polymorphonuclear cells were observed in control foreskin biopsies (Fig. 3A), which confirmed the absence of inflammatory response in those samples. Early PLS was characterized by hyperkeratosis of the epidermis, loss of dermal papillae, basal cell degeneration, dense inflammatory infiltration that contains of lymphocytes, macrophages, and monocytes which locate directly beneath basal cells layer and around blood vessels (Fig. 3B). Massive lymphocytic presence was also observed in moderate PLS while the epidermis with acanthosis and hyperkeratosis is separated by a narrow zone of hyalinization (Fig. 3C). On the contrary to early and moderate PLS, the severe stage is characterized by thin epidermis with hyperkeratosis, thick dermal hyalinization and sparse lymphocytic infiltrate in lower dermal layers (Fig. 3D). The predominance of connective tissue and the completed process of fibrosis in severe PLS seems to be the reason why the inflammatory process is weakly intensified at this stage. Observed high cytokines’ levels may correspond to numerous lymphocytes presented in sub-epidermal layers of dermis in early and moderate PLS (Fig. 3B, C). However, it does not correspond to sparse inflammatory cells in severe PLS (Fig. 3D).

### Cytokines’ gene expression levels in patients with micro-incontinence

**Fig. 4:**
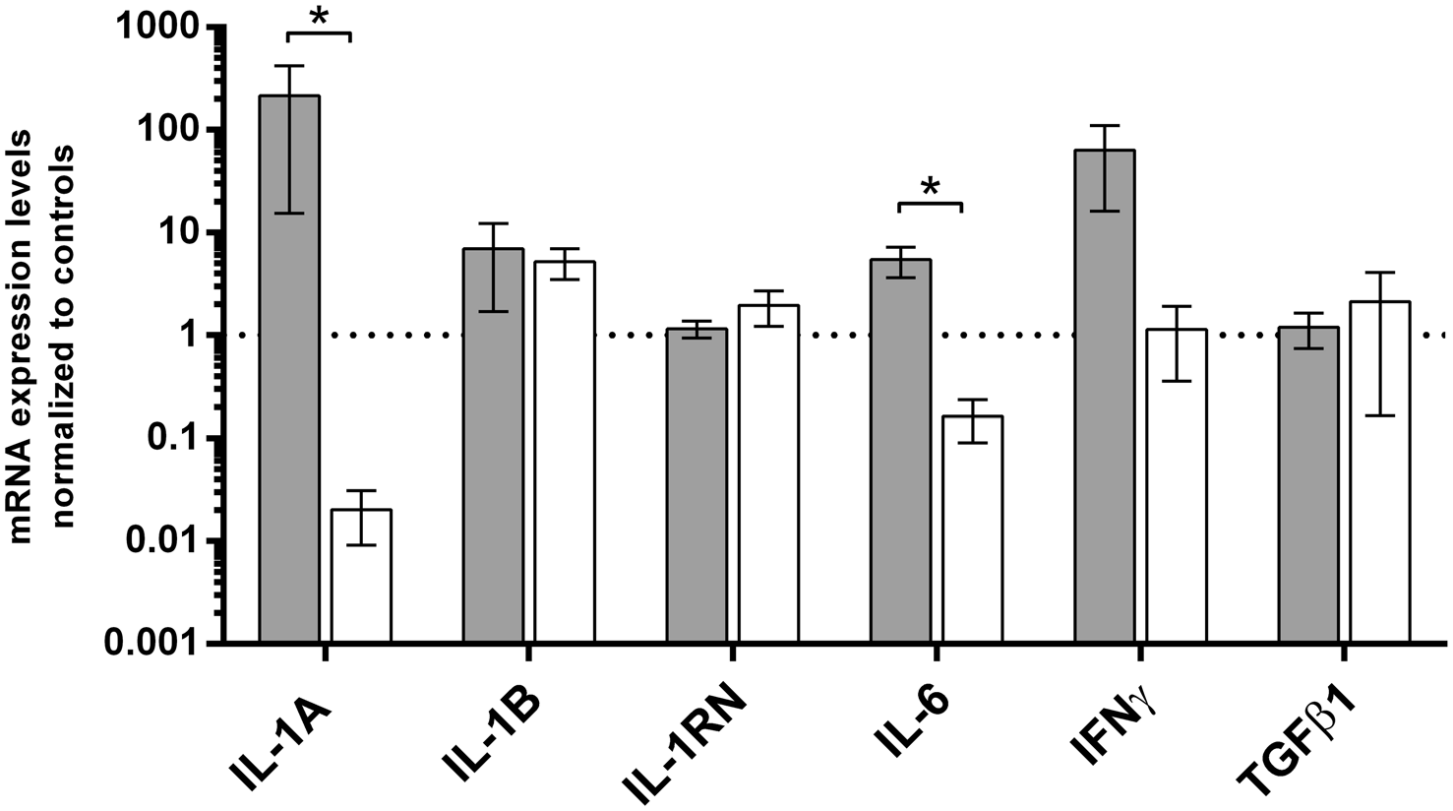
Summary of cytokines’ gene expression levels in penile lichen sclerosus to the occurrence of micro-incontinence. Gene expression was assessed as described in “Materials and methods”. The ordinate axis is shown on a logarithmic scale. Bars and whiskers represent the mean ± standard deviation of the mean (SEM) normalized to control foreskin samples (dotted horizontal line at 1), grouped by each analyzed gene. Bar legends: light grey bars represent PLS patients with micro-incontinence (MI) while white ones represent PLS patients without MI, respectively. *P < 0.05, Mann–Whitney *U* test between MI and lack of MI subgroups

The clinicopathological analysis showed that MI was highly prevalent in PLS since it occurred in 90% of patients (Table 3). Comparison of genes’ expression levels between MI and no MI subgroups revealed that IL-1A and IL-6 vs control ratios were ca. 400 and 30 times higher in PLS patients with MI than in PLS patients without MI, respectively (statistically significant ratios were noted for IL-1A and IL-6, respectively *P* = 0.0292 and 0.0144) (Fig. 4).

**Table 3:** Distribution of micro-incontinence in analyzed groups

Group	*N*	2 × 2 Fisher’s test of MI occurrence		CRP [mg/dL]: mean ± SD	P*	P** (all groups)	BMI	P*	P** (all groups)
		Versus control	Versus LS						
Control with MI	0								
Control no MI	13			1.76 ± 2.85		0.267	28.21 ± 6.96		0.74
LS MI	44	< 0.0001		4.834 ± 10.39	0.608		28.24 ± 3.81	0.11	
LS no MI	5			1.875 ± 1.601			24.05 ± 3.81		

*Mann–Whitney *U* test

**Kruskal–Wallis ANOVA test

## Discussion

Cytokines are small proteins secreted not only by cells of the immune system but also by many other cell types that play essential roles in autocrine, paracrine, and endocrine signaling in many organs and tissues. Interleukin 1 (IL-1A and IL-1B) is mainly pro-inflammatory and fibrogenic cytokine produced by macrophages, keratinocytes and endothelial cells [10]. IL-1 receptor antagonist (IL-1RN, alias: IL-1RA) acts antagonistically to IL-1; therefore, it has revealed anti-inflammatory properties [11]. This prevents the binding of the IL-1α and IL-1β to IL-1R1, which is a key step in the activation of the cascade of kinases that activates NF-kB and cyclooxygenase-2 (COX-2) [12]. Interleukin 6 is known as a pro-inflammatory cytokine secreted by monocytes and macrophages. However, IL-6 has a pleiotropic activity since it is secreted as acute-phase protein similarly to IL-1 [13]. TGF-β1 provides wound healing by promoting fibrosis [14]. INF-γ, apart from its antiviral activity, shows pro-inflammatory function by recruitment and activation of macrophages [15]. High immunoexpression of INF-γ was noted by immunohistochemistry (IHC) in inflamed regions of tissues of 12 vulvar LS [16] while increased levels of INF-γ, IL-4, TNF-α and IL-10 were observed in atopic dermatitis [17]. The mutual balance between cytokine production and expression of their receptors and antagonists was suggested to play a key role in the pathomechanism of lichen sclerosus characterized by initial inflammatory reaction followed by local fibrosis [16]. However, there is almost no data reflecting the expression of cytokines genes in the LS tissue what prompted us to determine the mRNA levels of *IL-1A, IL-6, IL-1B, IL-1RN, TGF-β1* and *INF-γ* genes in a large sample of penile lichen sclerosus tissue as compared to control penile tissues. Furthermore, we also analyzed the obtained data in the context of the micro-incontinence occurrence, and severity of lichen sclerosus.

Apart from one case report that presented one patient, our study is the first one, in which cytokines’ gene expression was analyzed in the penile tissue of lichen sclerosus patients [18]. There are studies concerning cytokines expression in vulvar LS. Farrell et al. checked IFN-γ, IFN-γ receptor, TNF-α, IL-1A, IL-2 receptor, intercellular adhesion molecule-1 (ICAM-1), and its ligand CD11a in vulvar LS specimens of 12 patients by immunohistochemistry (IHC). Interestingly, vulvar LS specimens have revealed higher expression of INF-γ than in morphologically normal vulva [16]. The LS specimens’ inflamed zone also showed increased immunostaining for TNF-α, IL-1A, IFN-γ receptor, IL-2 receptor, and ICAM-1 with its ligand CD11a. However, the fibrotic sclerosus zone in LS specimens had demonstrated reduced expression of INF-γ receptor, TNF-α, ICAM-1 and CD11a in comparison to inflamed fragments, but still higher than in normal vulva. Finally, ICAM-1 expression was higher in the epidermis of LS patients [16]. According to Corazza et al., in vulvular lichen sclerosus keratinocytes, and fibroblasts have revealed dysregulated cytokines expression compared with healthy skin, especially Chitinase-3-like Protein 1 (up-regulated), Growth Differentiation Factor-15 (up-regulated), Insulin-like Growth Factor Binding Protein-2 (down-regulated), and Dickkopf-related Protein-1 (down-regulated). Moreover, INF-γ and IL-8 were up-regulated in vulvar LS specimens [19]. These findings in female genital tissue confirm our observations of the increased expression, at the mRNA level, of pro-inflammatory cytokines in penile LS. Moreover, they are in line with our demonstration of a robust anti-fibrotic response from foreskin tissue with strongly increased expression of INF-γ, particularly in patients with early, and moderate PLS. The observed histological pattern of PLS tissue suggests that increased expression of cytokines’ mRNA implicates inflammatory cells as a possible source of cytokines in early and moderate PLS. However, the lack of infiltrating inflammatory cells in the penile dermis in severe PLS cases suggests that keratinocytes could be the main sources of at least some cytokines since they were shown in cultures to produce IL-1A and IL-1B. Indeed, biosynthesis of IL-1 family cytokines by keratinocytes was noted as a part of innate immunity response against bacterial infection. [20, 21] Secretion of IL-1A and IL-1B by keratinocytes was observed by Olaru et al. in an in vitro model of keratinocytes incubated with *S. aureus*.[20] Cheng et al. observed that infection with *C. trachomatis* leads to the secretion of IL-1A by keratinocytes [21]. Our report is the first that suggests the secretion of IL-1 family cytokines by keratinocytes as the result of the irritating effect of urine.

We found that micro-incontinence occurred more frequently in PLS (44 with MI vs. 5 who lack MI) than in control healthy males (0 vs. 13). Such observation leads to an obvious conclusion that the irritating urine effect on the prepuce’s susceptible epithelium may contribute to foreskin or penile disease. According to the study by Bunker et al. which involved 56 patients with penile LS, 94.6% of them reported post-micturition MI [2]. Doiron et al. reported that 16/19 obese patients with PLS acknowledged post-micturition MI [22]. Those reports are in line with our finding of nearly 90% MI incidence in LS cases. Prepuce, which covers the penile glans, is responsible for occlusion. Circumcision has been revealed as a successful treatment in males with LS in almost 100% cases [23]. Another study suggesting the role of occlusion and irritating effect of urine in PLS formation was carried out by Al-Niaimi et al. who studied 12 male patients with peristomal LS (11 patients with urostomy, and one with ileostomy). According to the zone of urinary bag, the circumferential occlusion was associated with the LS development what strongly suggests the role of skin irritation by urine in LS etiopathogenesis [24].

In such clinical-histopathological context of the studied patients with PLS, we suggest that irritating action of urine may stimulate the pro-inflammatory response of the penile skin mediated by cytokines. Indeed, we observed several hundred times higher levels of IL-1A mRNA and several dozen times higher IL-6 mRNA levels in PLS with MI than in few PLS patients without MI.

Our data confirm that occlusion and irritating urine effect provide increased expression of pro-inflammatory cytokines’ genes, i.e., *IL-1A* [10], *IFN-γ* [16], which probably take part in the development of penile LS, similarly to vulvar LS [16]. Our research is probably the first translational study trying to explain the pathomechanism of penile LS formation. However, there is a risk of bias caused by a relatively small number of patients. Moreover, in our study there were not compared the control group with urinary incontinence or PLS without occlusion. In addition, the involvement of cytokines was investigative at the level of mRNA expression, and we had not enough available tissues to assess the content of cytokines at the protein level, which should be the subject of future studies.

The main clinical conclusion of this study is to pay special attention to patients who report symptoms of micro-incontinence, and who additionally have problems with retracting the foreskin. We should either propose circumcision to such patients or place them under special scrutiny for the early signs of PLS.

## Conclusions

Occlusion and irritating urine effect are associative with highly increased expression of pro-inflammatory cytokines’ genes in foreskins that suggests a key role of IL-1A, IL-6 and IFN-γ in penile LS development and place them as possible new therapeutic targets.
